# The macroeconomics of COVID-19 exit strategy: the case of Japan

**DOI:** 10.1007/s42973-021-00091-x

**Published:** 2021-08-25

**Authors:** So Kubota

**Affiliations:** School of Political Science and Economics, Waseda University, 1-6-1 Nishiwaseda Shinjuku-ku, Tokyo, 169-8050 Japan

**Keywords:** COVID-19, SIR, Macroeconomics, Japan, Lockdown, Vaccine, E6, H1, I1

## Abstract

In this paper, I use a simple SIR Macro model to examine Japan’s soft lockdown policies in 2021 under the COVID-19 crisis. As real-time research, this paper consists of two parts written during two different research periods. The first part, which was originally reported in February 2021, studies the Japan’s second soft lockdown policy (state of emergency declaration) from January to March 2021. After the model is calibrated using 2020 data, the results show that a long enough lockdown can avoid future lockdowns, improving both the infection and the economy. In addition, I propose the ICU targeting policy, which keeps the number of severe patients at a constant level, mimicking the monetary policy’s inflation targeting. The model’s prediction is evaluated from an ex-post perspective in the second part, written in July 2021. I find that the model broadly captures the realized consequences of the second soft lockdown and the subsequent paths. Furthermore, the simulation is projected to the end of the pandemic under a revised scenario, incorporating the proliferation of COVID-19 variants. Finally, I discuss the effectiveness of the inverse lockdown (economic stimulus) policy in the fall of 2021 under the dynamic infection externality.

## Introduction

In reaction to the first wave of the COVID-19 pandemic, numerous countries imposed containment measures, such as curfews, school closures, and quarantines in the spring of 2020. The infection rate stayed at low levels over the summer, but most countries experienced their second or third waves in the autumn or winter. Many countries have returned to lockdowns to a greater or lesser extent, mainly due to their medical capacities. The spread of infection in some countries has been contained enough to lift such measures; however, governments remain concerned that a future wave of infections that will necessitate another round of lockdowns. Even though the COVID-19 crisis may be reaching its final stage, given the arrival of vaccines, containment policies remain adrift.

This paper aims to assess the policy implications of Japan’s 2021 lockdown policies on the economy and infection. The spread of COVID-19 in Japan has not been as extensive as in Europe and the United States, but the economic impacts have been comparable to those of these countries. In April and May of 2020, the Japanese government declared a state of emergency to stop the growing first wave of infections. This policy is called a *soft lockdown* or a *voluntary lockdown* (Watanabe and Yabu 2021a, b) since the restrictions are not as severe as the lockdowns in most countries.^1^ This paper will continue to refer to Japan’s policy as a soft lockdown for consistency. Contrary to the name, the Japan’s first soft lockdown significantly slowed the exponential increase of infection. However, like most other countries, Japan also experienced cyclical waves of infection, followed by soft lockdowns. In 2021, the Japanese government imposed the second lockdown from January to March, soon followed by the third lockdown from April to June.^2^

In this paper, I use a quantitative macroeconomic model incorporating epidemiological dynamics to study the consequences of Japan’s 2021 soft lockdowns on the economy and the spread of the virus. A novel aspect of this project is that the model and the policies are studied *twice* during different research periods. Usually, economic policy analysis are conducted after the policies have concluded as ex-post evaluations. This type of historical research is indeed valuable for gathering evidence and deriving suggestions for improving future policy designs. However, given that the circumstances of the COVID-19 crisis and the economy are rapidly changing day-to-day, there has been a growing demand for on-time policy research that builds on currently available information. One of the most successful projects in Japan is Fujii and Nakata (2021), who have been providing weekly updates of analyses on various policy issues using a SIR model that incorporates economic factors.^3^ My project is less frequent analyses, but it may still be worthwhile to summarize a trial of real-time analyses in this article.

For this purpose, this paper consists of researches conducted during two different time periods. The first part of the quantitative analysis (Section 4) was originally published in Kubota (2021) in *Covid Economics: Vetted and Real-Time Papers* in February 2021.^4^ I calibrated the model’s parameters using 2020 epidemic and economic data and then predicted the consequences of various policy options concerning the second soft lockdown, which was in progress at that time. The second part of the analysis (Section 5 and 6) was added in July 2021. From an ex-post perspective, I evaluated the predictive power of my model compared to the realized paths of both infection and economy. In addition, I conducted future prediction and policy analysis again with a revised scenario, including the future spread of COVID-19 variants.

The entire analysis is conducted using a simple SIR Macro model, following Eichenbaum et al. (2020a). This model includes agents’ optimizations of economic behaviors, which are in line with the empirical findings of the voluntary behavioral changes (Goolsbee and Syverson 2020; Watanabe and Yabu 2021a, b; Sheridan et al. 2020). I incorporate two factors into the SIR-Macro model. The first one is a decreasing trend of people’s subjective perceptions about COVID-19 infection, which is crucial to capturing Japan’s initial economic downturn in the spring of 2020 and sustaining recovery in the fall. The second is a sectoral division where one is associated with infections such as the face-to-face service, and the other is independent of virus transmission, such as online shopping. This model does a reasonable job of capturing both infection trends and economic dynamics during the first soft lockdown in April and May 2020 and the long-run trends throughout 2020.

In the February 2021 study, I quantitatively evaluate two policy options for the second soft lockdown, beginning in January 2021. The policy efficiency was measured according to the dominance relationship on the pandemic possibility frontiers, which describes the tradeoff between economic welfare costs and mortality rate, following Kaplan et al. (2020). It is a conservative policy evaluation method independent of normative judgment about the values of life.

The first policy exercise covers extensions of the second soft lockdown, which began in January 2021. If the government lifts this lockdown too early, the number of severely ill patients treated in the ICU will spike. Thus, the government needs to impose another lockdown, given the limited medical capacity. These recurrent lockdowns have been observed in many countries over the COVID-19 pandemic. The simulation shows that the government should extend the soft lockdown to sufficiently reduce infections, avoiding a subsequent lockdown until vaccines will be distributed. In SIR models, after lockdowns are lifted, the infections increase again. Repeated increases in infection rates, followed by recurrent lockdowns to reduce infection rates, have almost no overall impact on the pandemic (Moll 2020). Therefore, lockdown should be a one-time event, keeping the society safe until a vaccine is available for all.

I examine another policy, called ICU targeting, which keeps the number of severe patients treated in the ICU at a constant level. The concept is similar to inflation targeting in monetary policy, in which the policy instrument is the nominal interest rate, and the goal is to control the inflation rate. Under an ICU targeting policy, the policy tool becomes the method of containment, and the goal is to keep the number of ICU patients around the target. It is a variant of Miclo et al. (2020)’s filling-the-box strategy, designed to maintain ICU constraints until herd immunity is achieved. The ICU targeting policy can lead to less economic damage than the extensions of the soft lockdowns can attain under the capacity constraint. This is accomplished by keeping the ICU target close to the limit. However, the model results show that the ICU targeting policy is less efficient than a one-time lockdown of sufficient length, as ICU targeting tends to maintain behavioral restrictions for too long.

In July 2021, I evaluate my model from the ex-post perspective. After the government terminated the second soft lockdown on March 21, the infection rates exponentially increased again, partly due to the alpha variant. This prompted the Japanese government to impose the third soft lockdown from April 25 to June 20. Under these realized policies and vaccine distribution, with the parameters of the alpha variant calibrated from epidemiological reports, I compare the model’s simulated infection and economics paths, and the actual paths that occurred through June. The model broadly explains the realized paths: it captures the infection variables well. Still, it underestimates the number of deaths and consumption to some extent. On the other hand, the model’s economic variable is closely correlated with a mobility measure, although it is not considered in the February 2021 study.

I extrapolate the model to the future, where the herd immunity is obtained through vaccination. The model includes an extra acceleration of the pandemic caused by the delta variant, using parameters known as of July 2021. The delta variant significantly increases the number of ICU patients, but vaccinations of the elderly cancel it out. As a result, Japan may manage to converge the pandemic with no or limited containment measures. However, the model also predicts the substantial uncertainty in the infection and economic paths due to the delta variant’s parameter misspecification. In particular, the calibration error in the infection transmission parameter is more crucial than that of the severity.

As a final exercise, I quantitatively evaluate the inverse lockdown policy (Gonzalez-Eiras and Niepelt 2020), which boosts both economic activity and the rate of infection. This policy looks inefficient at first glance because the level of social activities is too high under the *static* externality. It is caused by people’s ignorance of COVID-19 transmission to other people in their decision makings. On the other hand, the inverse lockdown may be rationalized by *dynamic* externality (Garibaldi et al. 2020; Phelan and Toda 2021), in that social activities accelerate the spread of the pandemic to the point of herd immunity. Since people do not consider this, the government can improve economic efficiency by pushing them out of the home. In the early stages of the pandemic, when the infection rate grows exponentially, the static externality is also accelerated. However, during the convent phase of the pandemic under vaccine distribution, the dynamic externality is multiplied instead. Under the quantitative model with the realistic scenario, I numerically show that the inverse lockdown dominates and improves the economic welfare in the fall of 2021.

Related literature This research contributes to the rapidly growing literature of incorporating epidemiological SIR models into economics analysis (SIR Macro models). The basic structure of this paper’s model follows the work of Eichenbaum et al. (2020a). The formulation of the substitution between the two sectors is borrowed from Krueger et al. (2020). Subjective perception of the infection is also introduced in the work of Aum et al. (2020), von Carnap et al. (2020), and Hamano et al. (2020). In addition, there are many SIR-macro models focusing on time-varying optimal containment policies, age-dependent lockdowns, and testing and case-dependent quarantines.^5^ In addition, the ICU capacity constraint and its implications on lockdowns are studied by Miclo et al. (2020) and Moll (2020). Furthermore, the connections between dynamic externality, inverse lockdown, and vaccine arrival are explicitly or implicitly studied by Garibaldi et al. (2020), Gonzalez-Eiras and Niepelt (2020), Makris and Toxvaerd (2020), and Phelan and Toda (2021).

The SIR models are also applied to Japan. The closest to this paper is Hosono (2021), which applies a SIR Macro model to Japan’s first soft lockdown policy. The main difference between the models is that Hosono (2021) introduces the soft lockdown as a household preference change toward staying at home caused by government announcements, while this paper assumes a consumption tax on service goods. A few papers apply SIR models to Japan, but they omit agents’ optimizations on the tradeoff between health and economy.^6^ Fujii and Nakata (2021) and their regularly updated projects study various issues related to infection and the economy in Japan, such as soft lockdowns, vaccines, new variants, and the Tokyo Olympic game. In addition, Shibata and Kosaka (2021) study a SIR model with a time-varying infection parameter linked to a multi-sector econometric model. Based on an SIS model, Fukao and Shioji (2021) interprets the infection-economy tradeoff as the inflation-output relationship on the Philips curve and statistically tests the policy rule from the past data. There is another time-series econometric study by Tomura (2021), who considers various categories of consumption expenditures and their quantitative impacts on the effective reproduction number. Based on agent-based models, Chiba (2020, 2021) study the infection containment policies, such as contact-tracing apps, mobility control, shortening of restaurants’ opening hours, and working from home.

This paper is organized as follows. Section 2 introduces the SIR Macro model, and Section 3 provides the calibration of this model using 2020 data. The second soft lockdown policy is discussed in Section 4, which is written in February 2021. Section 5 extrapolates and evaluates the model in July 2021. The inverse lockdown is studied in Section 6. Finally, the conclusion appears in Section 7.

## Model

I extend the SIR-macro model presented in Eichenbaum et al. (2020a) to include two sectors, following the work of Krueger et al. (2020), and subjective perception about the infection, following studies by Aum et al. (2020), von Carnap et al. (2020), and Hamano et al. (2020).

### Economic environment

I consider a weekly model of discrete periods, $t = 0,1,2,\dots$. There is a unit mass of agents, and each maximizes the following discounted sum of utilities:

1 $$\begin{aligned} \sum _{t=0}^\infty \beta ^t \left[ \ln c_t - \theta \frac{n_t^2}{2} \right] , \end{aligned}$$

where $c_t$ is aggregated consumption and $n_t$ is hours of work. There are two types of goods: Good 1, which affects the infection such as face-to-face service good, and Good 2, including activities such as online shopping. The aggregated consumption $c_t$ is a bundle of two goods defined by the CES function with the elasticity of substitution $\eta$:

2 $$\begin{aligned} c_t = \left[ \frac{1}{2} (c_{1,t})^{1-1/\eta } + \frac{1}{2} (c_{2,t})^{1-1/\eta } \right] ^{\frac{\eta }{\eta -1}}. \end{aligned}$$

For simplicity, I assume the share of each good to be 1/2. As Krueger et al. (2020) emphasize, this two-sector assumption helps to capture the low infection rate in Japan, resulting from the substitution of Good 1 for Good 2. Moreover, this helps to explain the large drop observed in consumption under the first soft lockdown in April and May 2020.

The production of each good is linear in labor with the same productivity, *A*. Furthermore, the labor inputs are perfect substitutes between the two sectors; thus, the wage becomes constant. I normalize the wage as 1. The good markets are also perfectly competitive, and the prices of both goods are equal to the marginal productivity *A*.

### Infection

The infection follows the basic SIR epidemiology model. People are divided into four groups within each period *t*. The first one is susceptible at a mass of $S_{t}$, who are not yet infected but could potentially get sick in the future. The next one is infected at a mass of $I_{t}$, who are currently sick. After $I_t$, people enter the recovered group at a mass of $R_{t}$, or dead, a population of $D_{t}$.

Given the mass of new infections, $T_t$, each population evolves as

3 $$\begin{aligned} S_{t+1}&=S_{t}-T_{t}-\delta _t S_t, \end{aligned}$$

4 $$\begin{aligned} I_{t+1}&=I_{t}+T_{t}-\left( \pi _{r}+\pi _{d}\right) I_{t}, \end{aligned}$$

5 $$\begin{aligned} R_{t+1}&=R_{t}+\pi _{r}I_{t}+\delta _t S_t, \end{aligned}$$

6 $$\begin{aligned} D_{t+1}&=D_{t}+\pi _{d}I_{t}, \end{aligned}$$

where $\pi _{r}$ and $\pi _{d}$ are the recovery rate and death rate, respectively, and the fraction of vaccinated people among those susceptible is $\delta _t$. I assume the time-dependent rate to consider a realistic vaccination schedule in 2021.

I use superscripts *j* for each type: $j=s$ is for susceptible, $j=i$ for infected, and $j=r$ for the recovered. The allocation of each type *j* is a bundle of consumption and labor of Good 1 and Good 2, $\bigl ( (c^j_{1,t},c^j_{2,t}),(n^j_{1,t},n^j_{2,t}) \bigr )$. In this model, I assume that the mass of new infections depends only on the aggregate consumption of the susceptible and infected population. Specifically,

7 $$\begin{aligned} T_t = \pi _c \bigl ( S_t c^s_{1,t} \bigr ) \bigl ( I_t c^i_{1,t} \bigr ), \end{aligned}$$

where $\pi _c$ is the degree of infection through the economic interaction. This assumption is made for both simplicity and catching Japanese data. Eliminating infection through labor simplifies the equations of the dynamic system, whereas this assumption does not significantly alter the quantitative results. Regarding the data fit, the elimination of autonomous infection outside economic activities is used for magnifying the reduction of infection during Japan’s state of emergency in April and May 2020. One interpretation is that all social activities inevitably involve some level of spending.

The infection probability of each susceptible person consuming $c^s_{1,t}$ amount of Good 1 is represented by the function $\tau _t$ that

8 $$\begin{aligned} \tau _t(c^s_{1,t}) = \pi _c \bigl ( I_t c^i_{1,t} \bigr ) c^s_{1,t}, \end{aligned}$$

given the macro-level variables $I_t$ and $c^i_{1,t}$. The effective reproduction number in this model is defined as follows:

9 $$\begin{aligned} {\mathcal {R}}^0_t = \frac{T_t}{(\pi _r+\pi _d)(S_t+I_t+R_t)I_t}. \end{aligned}$$

### Decision problems

Susceptible To match Japan’s data, I introduce the susceptibles’ subjective perception about the total infected population, $I_t$. This subjective perception is represented as an exogenous variable $\psi _t$, which shows how much higher people believe the number of new infections is compared with the actual or reported number. That is, a susceptible person’s perception rate $\tau _t(c^s_{1,t})$ is replaced by $\psi _t \tau _t(c^s_{1,t})$ in her or his optimization problem. In the simulation, $\psi _t$ is initially assumed to be large because of people’s anxiety about the new coronavirus. As time goes by, however, people acquire better information, and then $\psi _t$ gradually decreases. This process follows a logistic function:

10 $$\begin{aligned} \psi _{t+1} = \psi _t - {\hat{\psi }} \cdot \psi _t^2 \cdot \left( 1- \frac{\psi _t}{\bar{\psi }} \right) , \end{aligned}$$

where $\bar{\psi }$ is the initial value equivalent to $\psi _0$, and ${\hat{\psi }}$ controls the speed of reduction. This perception factor $\psi _t$ is necessary to capture Japan’s large economic downturn under the backdrop of the small number of infections in March and April 2020. Moreover, the decreasing $\psi _t$ also traces out the recovery of consumption in the fall of 2020. A similar variable, called the fear factor, is also introduced by Aum et al. (2020) to capture the economic drop before in the United Kingdom and South Korea in the spring of 2020. von Carnap et al. (2020) assumes $\psi (t)$ to be a time-invariant parameter to explain the voluntary reduction of Uganda’s economic activities, and Hamano et al. (2020) discuss its implications for welfare-maximizing policies.

The following Bellman equation describes the optimization problem of each susceptible person:

11 $$\begin{aligned}&U_{t}^{s}=\frac{\eta }{\eta -1} \ln \left[ \frac{1}{2} (c^s_{1,t})^{1-1/\eta } + \frac{1}{2} (c^s_{2,t})^{1-1/\eta } \right] - \theta \frac{(n^s_t)^2}{2} \nonumber \\&\quad + \beta \left\{ \psi _t\tau _t(c^s_{1,t}) U_{t+1}^{i} + \delta _t U^r_t + \left[ 1-\psi _t\tau _t(c^s_{1,t})-\delta _t\right] U_{t+1}^{s}\right\} , \end{aligned}$$

where $U_{t}^{s}$ is the discounted sum of utilities of a susceptible person, and $U_{t}^{i}$ is that of an infected person. A susceptible person believes that he or she gets infected with probability $\psi _t \tau _t(c^s_{1,t})$ instead of $\tau _t(c^s_{1,t})$. If vaccines are distributed, she directly acquires immunization and joins $R_t$ with probability $\delta _t$. Each susceptible person maximizes her lifetime utility in Equation (11) under the budget constraint:

12 $$\begin{aligned} \left( 1+\mu _{t}\right) c_{1,t}^{s} + c_{2,t}^{s} = A n_{t}^{s} - B_t. \end{aligned}$$

The consumption tax rate of Good 1, $\mu _{t}$, represents Japan’s soft lockdown in this model, and $B_t$ is a lump-sum transfer. In reality, lockdowns are purely economic losses of capital and human resources. I assume that the tax revenue disappears under a violated government constraint with $B_t=0$. In this paper, I focus on economy-wide policies, where $\mu _{t}$ is independent of type $j=s,i,r$. The interpretation assuming this one sector shock to be lockdown may be debatable. In Western countries, strict lockdowns shut down almost all sectors, but the Japanese policy is a voluntary lockdown. The government asks for a reduction of operations in restaurants and bars, but many people still go outside to purchase necessities.

The optimality conditions for a susceptible person’s decision are obtained as follows:

13 $$\begin{aligned}&\frac{(c^s_{1,t})^{-1/\eta }}{(c^s_{1,t})^{1-1/\eta }+(c^s_{2,t})^{1-1/\eta }} = (1+\mu _{t}) \left( \frac{\theta }{A} \right) n^s_t + \beta \pi _c \psi _t \bigl [ (U_{t+1}^{s} - U_{t+1}^{i}) I_t c^i_{1,t} \bigr ], \end{aligned}$$

14 $$\begin{aligned}&\frac{(c^s_{2,t})^{-1/\eta }}{(c^s_{1,t})^{1-1/\eta }+(c^s_{2,t})^{1-1/\eta }} = \left( \frac{\theta }{A} \right) n^s_t . \end{aligned}$$

Infected The problem of an infected person is much simpler because this person will not become reinfected. Each infected person solves the following equation:

15 $$\begin{aligned}&U_{t}^{i}=\frac{\eta }{\eta -1} \ln \left[ \frac{1}{2} (c^i_{1,t})^{1-1/\eta } + \frac{1}{2} (c^i_{2,t})^{1-1/\eta } \right] - \theta \frac{(n^i_t)^2}{2} \nonumber \\&\quad + \beta \left[ \pi _{r} U_{t+1}^{r} + \pi _d \times 0 + \left( 1-\pi _{r}-\pi _{d}\right) U_{t+1}^{i}\right] \end{aligned}$$

16 $$\begin{aligned}&\text {s.t. } \left( 1+\mu _{t}\right) c_{1,t}^{i} + c_{2,t}^{i} = A n_{t}^{i} - B_t. \end{aligned}$$

An infected person will be recovered with probability $\pi _{r}$ and obtain the discounted sum of utility $U_{t+1}^{r}$. The individual value of death is normalized as 0, following Eichenbaum et al. (2020a). With probability $1-\pi _{r}-\pi _{d}$, such as a person remains as infected. For simplicity, I do not assume a labor productivity decline due to infection, which makes the dynamic system drastically simple, as shown by Krueger et al. (2020). The first-order conditions are

17 $$\begin{aligned}&\frac{(c^i_{1,t})^{-1/\eta }}{(c^i_{1,t})^{1-1/\eta }+(c^i{2,t})^{1-1/\eta }} = (1+\mu _{t}) \left( \frac{\theta }{A} \right) n^i_t, \end{aligned}$$

18 $$\begin{aligned}&\frac{(c^i{2,t})^{-1/\eta }}{(c^i{1,t})^{1-1/\eta }+(c^i{2,t})^{1-1/\eta }} = \left( \frac{\theta }{A} \right) n^i_t . \end{aligned}$$

Contrary to the susceptible person’s problem, the choice of $c^i_{1,t}$ does not affect future values. Given the two first-order conditions and the budget constraint, the infected person chooses the allocation $(c^i_{1,t}, c^i_{2,t}, n^i_t)$.

Recovered Finally, the decision problem of each recovered person is similarly defined as

19 $$\begin{aligned}&U_{t}^{r}=\frac{\eta }{\eta -1} \ln \left[ \frac{1}{2} (c^r_{1,t})^{1-1/\eta } + \frac{1}{2} (c^r_{2,t})^{1-1/\eta } \right] - \theta \frac{(n^r_t)^2}{2} + \beta U_{t+1}^{r} \end{aligned}$$

20 $$\begin{aligned}&\text {s.t. } \left( 1+\mu _{t}\right) c_{1,t}^{r} + c_{2,t}^{r} = A n_{t}^{r} - B_t. \end{aligned}$$

A recovered person retains this recovered status. As the infected person’s problem, Good 1 consumption of the recovered person, $c^r_{1,t}$, is also independent of future values. Next, as in Krueger et al. (2020), the allocation of a recovered person becomes the same as that of an infected patient:^7^$(c^i_{1,t}, c^i_{2,t}, n^i_t) = (c^r_{1,t}, c^r_{2,t}, n^r_t)$. Therefore, this model’s dynamic system includes only $(c^i_{1,t}, c^i_{2,t}, n^i_t)$.

### Equilibrium

Given the perfect substitution of labor inputs between Sector 1 and 2, the equal linear labor productivity, and $B_t=0$, the equilibrium conditions of both goods are integrated into

21 $$\begin{aligned} (1+\mu _t) S_t(c^s_{1,t}+c^s_{2,t}) + (1+\mu _t)(I_t+R_t)(c^i_{1,t}+c^i_{2,t}) = A S_t n^s_t + A (I_t+R_t) n^i_t. \end{aligned}$$

It is redundant^8^ given the budget constraints of the three types: Eqs. (12), (16), and (20).

Finally, the dynamic system of the equilibrium equations is summarized by

$$\begin{aligned} {\left\{ \begin{array}{ll} \text {15 variables: } &{} c^s_{1,t}, c^s_{2,t}, n^s_t, c^i_{1,t}, c^i_{2,t}, n^i_t, \tau _t, T_t, S_t, I_t, R_t, D_t, U^s_t, U^i_t, U^r_t \\ \text {15 equations: } &{} (3); (4); (5); (6); (7); (8); (11); (12); (13); (14); (15); (16); (17); (18); (19):.\end{array}\right. } \end{aligned}$$

given the exogenous path of $\psi _t$ following Equation (10), and exogenous shocks of $\mu _t$ and $\delta _t$.

## Calibration and model’s evaluation

### Calibration

I use Our World in Data COVID-19 database maintained by Max Roser and Hasell (2020) for infection. The daily data are summed up on a weekly basis, and the consumption statistics are taken from the Survey of Household Economy. I use a seasonally adjusted monthly series, normalize the level as 1 in January 2020, and convert to weekly data through linear interpolation. The estimated effective reproduction number is taken from Toyo–Keizai Online.^9^ Following Eichenbaum et al. (2020a), the discount factor $\beta$ is $(0.96)^{1/52}$. I assume 18 weeks for average infection periods. At the end of 2020, the number of total deaths in Japan is 3292 out of 235811 total cases. By $0.014 = 3292/235811$, I set^10^$\pi _d = (7/18) \times 0.014$, and $\pi _r = (7/18) \times [1-0.014]$. The elasticity of substitution is assumed to be $\eta = 3$ from the lower case number of Krueger et al. (2020).^11^ Next, *A* and $\theta$ are calculated from the equations in the pre-pandemic steady state, when all people are susceptible and $c^1_{s,0} = c^2_{s,0} = 1/c_{s,0}$. In the Survey on Time Use and Leisure Activities in 2016, the average hours of paid work are 241 minutes among the entire population over age 10. Following this, weekly hours of work in the pre-pandemic steady state are $n_{s,0} = 241 \times 7 / 60$. From the World Bank data, the Japanese GDP per capita in 2016 is $52 \times c_{s,0} = 39289$ to the US dollar. Then, $A = c_{s,0}/n_{s,0} = 26.8729$. The labor disutility weight $\theta$ is obtained from the pre-pandemic steady-state condition $\theta = 1/(n^s_{0})^2 = 0.001264$, which is derived from Eqs. (12), (13), and (14).

The exogenous path of the perception rate, $\bar{\psi }$ is calibrated to roughly capture the observed reduction of consumption before the soft lockdown in April and May 2020. Moving forward, ${\hat{\psi }}$ is decided so that $\psi _t$ becomes about 1 at the end of 2021. It is reasonable that people perceive the infection rate almost correctly around the end of the pandemic. I choose^12^$\bar{\psi }=15$ and ${\hat{\psi }}=0.015$. Finally, I set $\pi _c = 0.00000416$ to roughly match the total number of deaths at the end of 2020.^13^ To explain the consumption drop during the first soft lockdown, from the second week of April until the third week of May 2020, I set $\mu _{t} = 0.35$.

This simulation begins from the exogenous initial infection shock $I_1 = 0.00001$ in the second week of January.^14^ This economy follows the perfect foresight path until it converges to the new steady state in 250 weeks.^15^

### Japan’s COVID-19 infection and economy in 2020

**Fig. 1:**
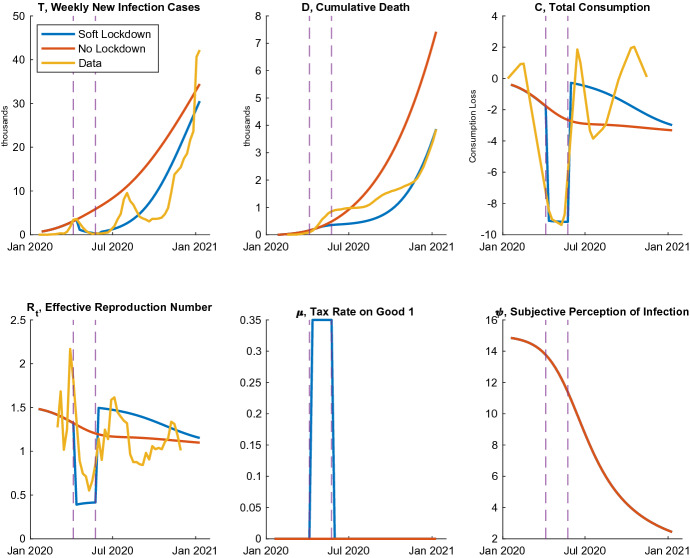
Infection and economy in 2020

Figure 1 shows the simulation results both with and without the soft lockdown and data in 2020. Given the only two exogenous variables $\mu _t$ and $\psi _t$, the simulation with the first soft lockdown captures both the infection and economic paths of Japan from January to December 2020. In addition, this model explains the impacts of the soft lockdown in April and May on new infections, consumption, and the effective reproduction number. Beyond this, the model shows the number of cumulative deaths at the end of 2020. However, it fails to describe the second wave of infection and the short-term fluctuation of consumption in the summer and fall of 2020. This may be caused by the cash transfer policy called the Special Fixed Benefits or the subsidy for travel called the “GoTo Travel” program. I do not include these factors in the model to concentrate on analyzing the soft lockdown and avoid risks due to the uncertain quantitative impacts of these policies.^16^ Additionally, this simulation implies that, if there had been no soft lockdown, the cumulative death total would have been nearly twice as high in 2020.

### Medical environment for policy analysis in 2021

Beyond the calibration using 2020 data, I introduce the ICU capacity constraint and vaccine plans. They are redundant in 2020 but crucial for the policy exercise in 2021.^17^

The ICU Capacity Constraint The total number of severely ill patients must be below the maximum level that the available medical facilities can accommodate. In January 2021, during the second soft lockdown, the actual number of ICU patients was about 1000. Although Japan’s official total ICU capacity is 3600, hospitals in urban areas had difficulty accepting severe patients needing immediate treatment. Given these conditions, I set Japan’s ICU capacity constraint at 1200.

Because the model does not explicitly include the stage of severe illness, I calculate the number of ICU patients in simulation from the observed relationship between the number of patients and the number of deaths in data. Using the nonlinear least square regression for a quadratic equation using the data between the fourth week of October and the second week of January, I obtain

22 $$\begin{aligned} ICU_t = 0.66506*(D_t-D_{t-1}) + 636620*(D_t-D_{t-1})^2, \end{aligned}$$

where $ICU_t$ is the number of ICU patients in Week *t* and $D_t-D_{t-1}$ is the number of new deaths given the normalized population 1. The constant term is omitted because $ICU_t = D_t=D_{t-1}=0$ in the pre-pandemic steady state.

Two Vaccine Scenarios The new coronavirus infection eventually disappears due to the introduction of a vaccine in 2021. Thus, I conduct policy exercises under the following opportunistic and pessimistic scenarios.

- **Vaccine 1**: This is an opportunistic scenario, following the government’s ideal vaccine distribution plan as of January 2021.^18^ In the first week of April, the vaccine administration to the elderly and people with underlying conditions begins. Given that each vaccine requires two shots with a three-week interval, they begin to get immunized in the third week of April. The government finishes their second shots at the end of June, and then immunization begin for other people. Because the total elderly population is about 36 million, I assume 4 million people obtain immunization per week. Moreover, the vaccination rate continues to increase; that is, 4 million people get vaccinated after July as well. As a result of the vaccination of the elderly, the death rate declines from 0.014 to 0.0035 between the first week of April and the end of June.^19^
- **Vaccine 2**: This is a relatively pessimistic but realistic scenario roughly following Fujii and Nakata (2021). It is based on the evidence of countries showing when vaccination begins in Japan and the observed delays from their original plans. As in the opportunistic scenario, people begin getting immunized during the third week of April. The weekly number of people obtaining immunization linearly and gradually increases from 0.1 million in the third week of April to 1.6 million at the end of June. After that, the weekly number stays constant at 1.6 million. The elderly become immunized beginning in the third week of April, and it takes 23 weeks (until the last week of September) for $80\%$ of them to acquire immunity. In these 23 weeks, the death rate linearly declines from 0.014 to 0.0035, as in the opportunistic case.

## Policy exercise in 2021

In this section, I consider two policies following the second soft lockdown originally planned to be lifted in the first week of February. The first one is extending the soft lockdown with the same degree of stringency. Under this policy, if the government stops the behavioral restrictions too early, it will need to declare one more lockdown due to the ICU capacity constraint. The second case is beginning a new policy during the second week of February that keeps the number of ICU patients at a constant level, below the ICU capacity.

### Extending soft lockdown

The first policy I consider is a prolonged soft lockdown, starting in January. By matching the model and the observed number of new infections at the end of January, I calculate the second soft lockdown’s tax rate as half of the first one, $\mu _{t} = 0.175$. The government maintains the same stringency in the extended periods as well. If the government lifts the second lockdown quickly, another one will be required to maintain the ICU capacity constraint. I assume that the government imposes a four-week lockdown with $\mu _{t} = 0.175$ if the patients fill more than $70\%$ of the ICU capacity. In the simulation, this simple rule keeps the medical capacity at a favorable margin.

**Fig. 2:**
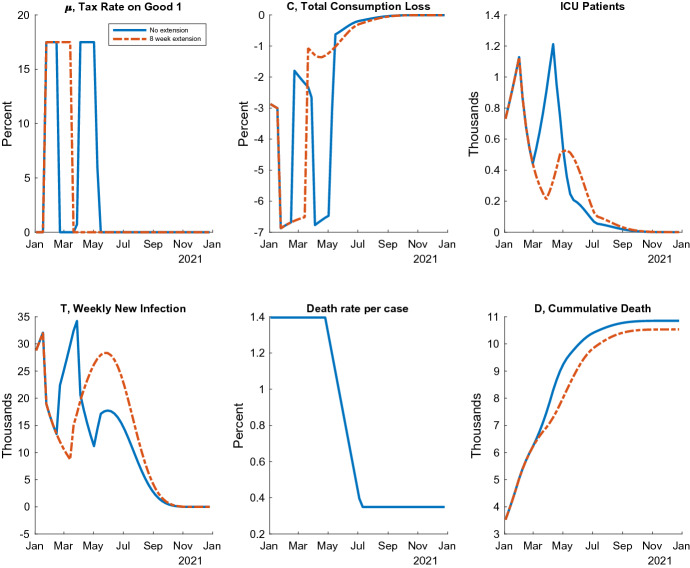
Extending the soft lockdown under Vaccine 1

Two Examples of Equilibrium Paths Fig. 2 shows two examples of the equilibrium paths in 2021 with Vaccine 1 for illustration. One is a short soft lockdown lifted in the first week of February, as the original government policy plan, and the other is a long lockdown with and 8-week extension. In the first case, the number of ICU patients increases after the end of soft lockdown and reaches the 1200 ICU capacity constraint in April. Next, the government imposes one more lockdown for 4 weeks. The consumption almost fully recovers in the summer of 2020 because the risk of infection declines due to lockdowns and vaccines. In the second case, the soft lockdown in January stays the infection low enough to avoid filling all the ICU beds. The consumption also recovers quickly. A key feature of this plan is that the number of new infections increases drastically in the summer, while the number of ICU patients drops down due to vaccination among the older population. By allowing the virus to flourish among the young, the economy quickly recovers but limits the number of deaths. These combinations of a lockdown before the vaccine and no restriction after could be effective. They implicitly implement age-dependent policies, which significantly reduce the economic costs while keeping the high-risk elderly safe (Acemoglu et al. 2020; Favero et al. 2020).

Pandemic Possibility Frontiers These policies are evaluated using the dominance relationship in terms of both health and economic damages on the pandemic possibility frontiers, following Kaplan et al. (2020). Specifically, I illustrate a tradeoff between the number of total deaths at the end of 2021 and the economic welfare costs of living people in 2021.^20^ I measure the latter as the consumption equivalence, which is defined as the solution *x* to the following equation:

23 $$\begin{aligned}&\left[ \sum _{\tau =0}^{52} \beta ^\tau \right] \left[ \ln \bigl ( c_0 (1-x) \bigr ) - \theta \frac{(n_0)^2}{2} \right] \nonumber \\&\quad = \sum _{\tau =0}^{52} \beta ^\tau \left\{ \frac{ S_\tau \left[ \ln \bigl ( c^s_{\tau } \bigr ) - \theta \frac{(n^s_{\tau })^2}{2} \right] + (I_\tau +R_\tau ) \left[ \ln \bigl ( c^i_{\tau } \bigr ) - \theta \frac{(n^i_{\tau })^2}{2} \right] }{ S_\tau + I_\tau + R_\tau } \right\} , \end{aligned}$$

where $c_0$ and $n_0$ are the pre-pandemic steady state total consumption and labor, respectively, and the period $\tau$ is normalized as the first week of January 2021.

**Fig. 3:**
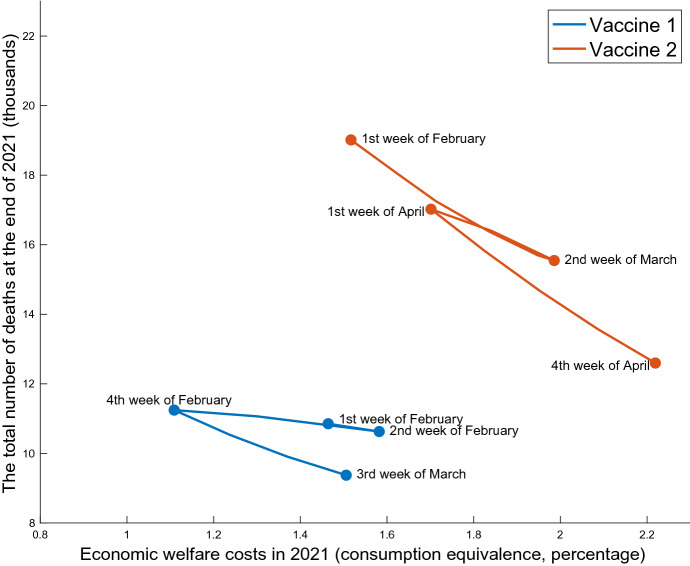
Pandemic possibility frontier under the extension of soft lockdown

Figure 3 shows the frontiers of the soft lockdown extensions with Vaccine 1 and Vaccine 2. These two are quantitatively different due to differing speeds of vaccine distribution, but their qualitative implications are similar. In particular, both show the inefficiency of recurrent lockdowns. If the soft lockdown lifts before the fourth week of February under Vaccine 1 or the first week of April under Vaccine 2, the government will need to impose one more lockdown given the ICU capacity constraint. Next, sufficiently long lockdowns achieve both lower economic losses and fewer deaths than the recurrent cases in certain regions on the diagram. In general, lockdowns are similar to time machines; that is, they push the state of infection back to the level before the policy. In other words, the infection rate similarly grows again after lifting lockdowns. The primary role of lockdowns is not eliminating the entire pandemic but postponing the exponential increase of infections to allow for the arrival of vaccines. Thus, if there is a repeating expansion and contraction of infections caused by recurrent lockdowns, the time machine just goes to the past and comes back. It has almost no impact on the spread of the new coronavirus or on the economy. Therefore, the lockdowns should be one-time event to keep the number of ICU patients below the capacity until the arrival of vaccine.

### ICU targeting

**Fig. 4:**
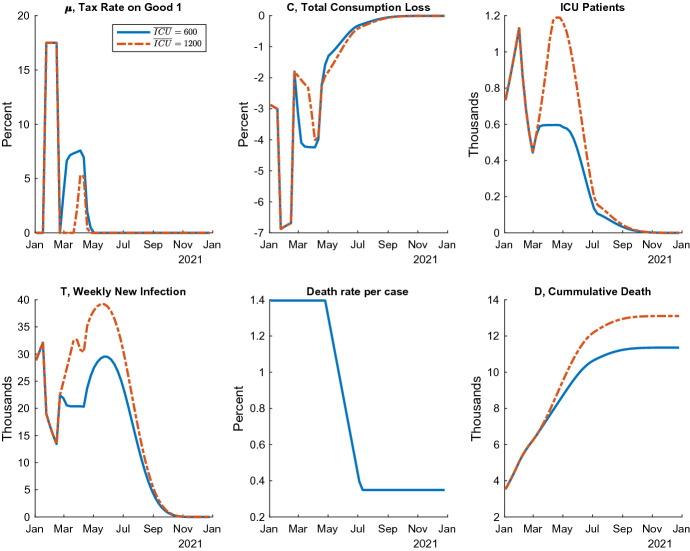
ICU targeting under vaccine 1

Next, ICU targeting is another policy rule that keeps the number of patients in the ICU at a constant level ${\overline{ICU}}$ below the 1200 capacity. To keep this target, the government flexibly adjusts the tax rate $\mu _{t}$. This idea is similar to the inflation targeting in monetary policy. In many countries, central banks adjust the nominal interest rates to achieve the target rate of inflation. In ICU targeting, the policy goal is changed to the number of severe patients, and the policy tool becomes the degree of the restrictions.

In my scenario, the government lifts the soft lockdown in the first week of February according to the original plan and changes the policy rule to ICU targeting, beginning from the second week of February. To keep the number of ICU patients $ICU_t$ around the target ${\overline{ICU}}$, the government adjusts the tax rate following the equation below:

24 $$\begin{aligned} \mu _{t} = \min \left\{ 0.1, \ \frac{40}{({\overline{ICU}})^2} \bigl [ \max \{ 0, \ ICU_t - 0.95 \cdot {\overline{ICU}} \} \bigr ]^2 \right\} . \end{aligned}$$

This equation means that the tax rate $\mu _{t}$ increases from $0\%$ to $10\%$, while $ICU_t$ increases from $5\%$ below ${\overline{ICU}}$ to ${\overline{ICU}}$. If $ICU_t>{\overline{ICU}}$, the tax rate $\mu _{t}$ is constant at $10\%$. Although this policy does not precisely maintain the $ICU_t$ at ${\overline{ICU}}$ exactly, it reasonably achieves the goal. I do not impose the exact targeting to avoid the critical non-linearity in the computation.

Two ICU targeting examples Figure 4 describes two equilibrium paths under ICU targeting policy, where ${\overline{ICU}} = 600$ and ${\overline{ICU}} = 1200$. The latter decides the target at the capacity. The tax rate $\mu _{t}$ is flexibly adjusted to keep the number of severe patients at 600 or 1200 in the spring of 2021. Because of the rapid decline of the death rate in the summer, the government ends the behavioral restrictions, allowing $ICU_t$ to decline. As in the case of the extension of the soft lockdown, the rise in the number of new infections in May and June implements an age-dependent policy. In addition, the consumption quickly recovers in the summer while limiting deaths.

**Fig. 5:**
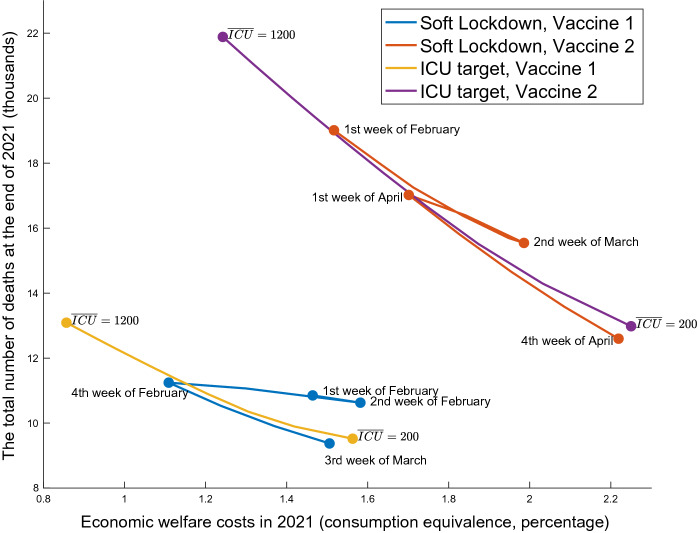
Pandemic possibility frontiers, including ICU targeting

Pandemic possibility frontiers Figure 5 shows the pandemic possibility frontiers under the ICU targeting policies in Vaccines 1 and 2, as well as those of soft lockdown extensions, which appear in Fig. 3 for comparison. From the upper left to the bottom right, I move the target level from the maximum 1200 down to 200 and illustrate the consequences of economic loss and death as a locus. Thus, the ICU targeting policy can achieve less economic damage that is not obtained by the soft lockdown. The ICU targeting is necessary to push the economic welfare costs lower than about $1.1\%$ under Vaccine 1 or $1.5\%$ under Vaccine 2. This is achieved if the Japanese government keeps the number of ICU patients close to the limit. This can be justified if the society values the life less than a certain level.^21^ However, the ICU targeting is inferior to the one-time prolonged lockdown, in which the time of lifting is between the fourth week of February and the third week of March under Vaccine 1 and the first week of April and the fourth week of April under Vaccine 2. This is because the ICU targeting tends to continue restrictions for too long after the start of vaccination, which distorts the young’s economic activities.

The pandemic possibility frontiers also illustrate substantial economic and health benefits by hastening the vaccine distribution. If the Japanese society chooses the economic damage as $1.5\%$ of the consumption under both vaccine cases, the number of deaths can be reduced from about 19, 000 with Vaccine 2 to 9, 000 with Vaccine 1. On the other hand, if the number of deaths is fixed at 13, 000, the economic damage can be reduced from about $2.2\%$ to less than $0.9\%$ in the consumption. For comparison, Japan’s total budget of both the central and local governments for the vaccine distribution is about only $0.25\%$ of the GDP.^22^ By accelerating only the vaccine supply, the economic damage can be improved by $1.3\%$ of the consumption. Acharya et al. (2020) estimate a greater economic value by studying stock price reactions to the development progress indicator and yielding an even higher value.

## Ex-post evaluation

This section was written in July 2021. From an ex-post perspective, I evaluate my quantitative model constructed in the previous sections.

### The scenario

The Japanese government lifted the second soft lockdown on March 21 and imposed the third soft lockdown from April 25 to June 20, except for Okinawa Prefecture. According to Fig. 5, the model predicts the third soft lockdown based on the speed of vaccine distribution. The third soft lockdown could have been avoided if vaccine distribution followed the original government plan, Vaccine 1. In contrast, the third soft lockdown was necessary under Vaccine 2. The actual path fell between these two cases and therefore the third lockdown was partially consistent with the model’s prediction.^23^ Furthermore, the Japanese government started the fourth one only in Tokyo (and continued in Okinawa) from July 12.

For the evaluation of my model and future prediction, I need to decide the stringency $\mu _t$ of the second, third, and fourth lockdowns. For this purpose, I employ the Community Mobility Reports of Google. Using the several measures included in the reports, I make a weekly mobility index as the average of parks, transit stations, retail and recreation, and workplace measures. Next, I estimate the relationship between this mobility index and the observed weekly consumption from the Family Income and Expenditure Survey between March and December 2020. By the nonlinear regression, it is estimated as

$$\begin{aligned} Mobility_t = 1.52 \times Consumption_t - 19.52 \times (Consumption_t)^2. \end{aligned}$$

The constant term is eliminated because both indices should be zero in the pre-pandemic steady state. Then, given an assumed value of $\tau _t$ in the periods of the soft lockdowns in 2021, the model’s simulation generates the mobility index using this equation. I calibrate $\tau _t$ so that the average mobility index generated by the model in each soft lockdown matches the observed one from the Community Mobility Reports of Google in the same periods. As a result, I obtain $\mu _t = 0.197$ for the second lockdown, and $\mu _t = 0.158$ for the third one. However, I assume $\mu _t = 0$ for the fourth one because it is imposed only in Tokyo and Okinawa Prefectures, and the mobility index has not declined at the national level as of July.

Other primary triggers of the 2021 soft lockdowns were COVID-19 variants. The infection caused by the alpha variant (lineage B.1.1.7) significantly increased in March and April in the Kansai region, and others soon followed. According to National Institute of Infectious Diseases (2021), as of the beginning of June, more than 90% of COVID-19 cases consisted of the alpha variant. The infection rate of the alpha variant is estimated to be 32% higher. The alpha variant may also increase the severity as reported in other countries. As Horby et al. (2021) summarize, there is a large uncertainty among estimates by various studies. I decide to follow Davies et al. (2021), which has the largest sample size, and choose the $61\%$ increase in the death rate $\pi _d$.

In addition, the delta variant (lineage B.1.617.2) began to spread, and by the third week of June, its share of COVID-19 cases increased to 8.2% in Tokyo.^24^ A Japanese report suggests that the delta variant will almost perfectly replace other variants between June and August.^25^ It is consistent with the evidence of the United Kingdom, where the delta variant rapidly spread and became dominant in April and May. However, the transmission rate is highly uncertain. Public Health England at first estimated the secondary attack rate of the delta variant to 35% higher than that of the alpha variant; however, they later reported that the difference significantly shrank. I follow their latest report (Public Health England 2021) and assume that the transmission rate of the delta variant as $3.5\%$ higher than that of alpha, as the baseline. They also estimate that the hazard rate of emergency care attendance due to the delta variant is 80% higher than the alpha variant in Scotland. I use this number for the increase in the death rate $\pi _d$.

Medical experts also concern about the decline in vaccine effectiveness caused by the spread of the variants. Lopez Bernal et al. (2021) reported that the effectiveness of full vaccination of BNT162b2 (Pfizer) and ChAdOx1 (AstraZeneca) for the alpha variant are 93.7& and 74.5%, respectively, and those for the delta variant are 88.0% and 67.0%.^26^ Although the evidence is limited as of July 2021, I set the effectiveness as 0.8 for this simulation.

Given this information, I consider the following scenario for evaluating my model built in February 2021 and future predictions as of July 2021.

- The second soft lockdown continues from the second week of January to the third week of March. The tax rate on the Good 1 is 19.7%.
- The third soft lockdown continues from the fourth week of April to the third week of June. The tax rate on the Good 1 is 15.8%.
- A fourth soft lockdown or more is not assumed.
- Immunization begins the third week of April. The weekly number of people receiving immunizations gradually increases from 0.1 million the third week of April to 4 million by the end of July. Vaccinations of the elderly are completed by the end of July, as the Japanese government plan as of June 2021. After that, the weekly number of vaccinations stays constant at 4 million. Vaccinations end the first week of November when 80% of the total population finished full shots of vaccine. Furthermore, given the vaccine effectiveness, the actual number of people who obtain immunization is reduced by 20%. In the model, the vaccination parameter $\delta _t$ is uniformly declined by 20%. As a result, 64% of the total population obtain immunization at last.
- Following vaccination of the elderly, the baseline death rate $\pi _d$ will decline from 0.014 to 0.0035 by the end of July. This baseline rate can change depending on the spread of the variants.
- The alpha variant’s share of infections linearly increases from the first week of March and then reaches 100% the fourth week of May. This pushes up the infection parameter $\pi _c$ by 32% and the death rate $\pi _d$ by 61%.
- The delta variant’s share of infection linearly increases from the second week of June. It reaches 100% in the third week of August. It pushes up the infection parameter $\pi _c$ by 3.5% and the death rate $\pi _d$ by 80% in addition to the alpha variant.

### Simulation

**Fig. 6:**
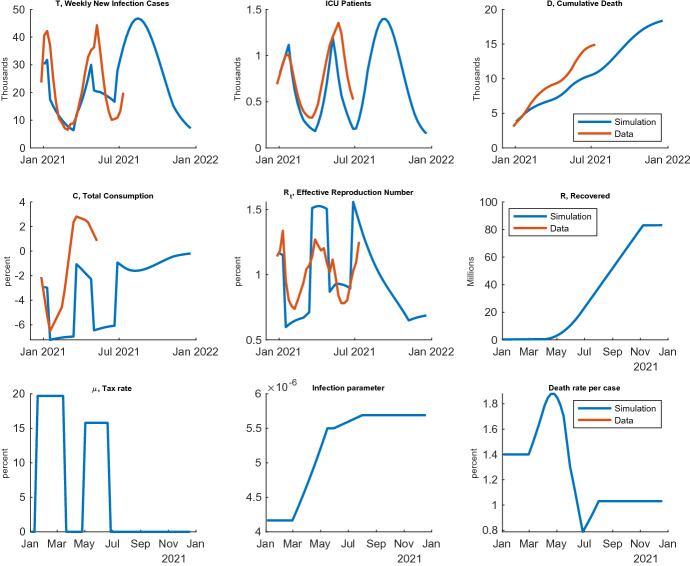
Simulation in 2021 with lockdowns and variants

Figure 6 plots the baseline simulation results. Overall, the simulation captures the fluctuation of new cases and the number of ICU patients. The effective reproduction number is also roughly consistent. However, this simulation, to some extent, underestimates cumulative death. Given the good fit of ICU patients, the model may fail to include the decline in medical system efficiency due to ICU congestion.

The infection parameter $\pi _c$ increases with the alpha and delta variants, significantly raising the number of new infections after the third lockdown, as in the bottom middle diagram of Fig. 6. These two variants also push up the death rate $\pi _d$; however, this rate declines with vaccination of the elderly, as shown in the bottom right diagram. As a result, the increase in the number of ICU patients is limited. That is, the Japanese government may not need additional soft lockdowns until the herd immunity. Indeed, this prediction is not robust to the parameter assumption. Given possible uncertain factors, including the Tokyo Olympic event, the government’s decision of additional lockdowns, such as the fourth lockdown of Tokyo, may be reasonable.

**Fig. 7:**
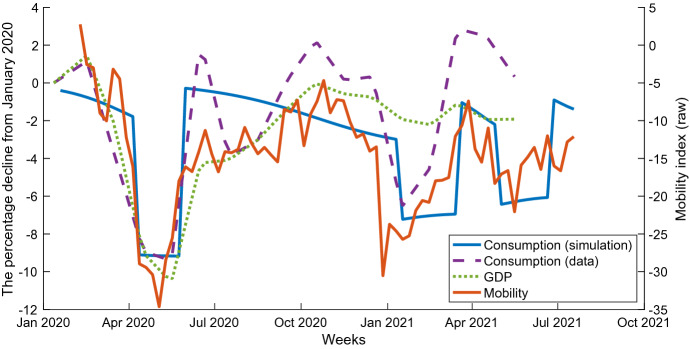
Simulation and three economic measures

About the economic side, the model fails to capture the significant increase in total consumption in the spring of 2021. Since this jump exceeds the pre-pandemic level, my model is impossible to capture whatever the scenario and parameters are. In Fig. 7, I compare my simulation results with the consumption measure of the Family Income and Expenditure Survey, Community Mobility Reports of Google, and monthly GDP estimates provided by The Japan Center for Economic Research (JCER).^27^ In 2020, the three measures were consistent, while consumption fluctuates more. However, in the spring of 2020, consumption jumps up substantially higher than the other two economic measures. In contrast, the GDP shows almost no decline responding to the second soft lockdown. Under my model, it is insufficient to explain the decline in the number of infections. My model’s consumption result is highly consistent with the mobility index. Compared to the other two, it is directly associated with the infection. Moreover, since the mobility can be directly observed from the cell-phone location data, it may be robust to estimation errors.

The economic data under the COVID-19 crisis suffer from significant measurement problems. Since the COVID-19 crisis is a biased shock to the service sector, the composition of goods and relative prices significantly fluctuated. It makes the measurement of aggregate level variables hard. For instance, Blundell et al. (2020) and Abe et al. (2020) point out the measurement bias in the consumer price index (CPI). Moreover, since the period of economic cycles is about a few months, it is hard to eliminate the seasonality.^28^

**Fig. 8:**
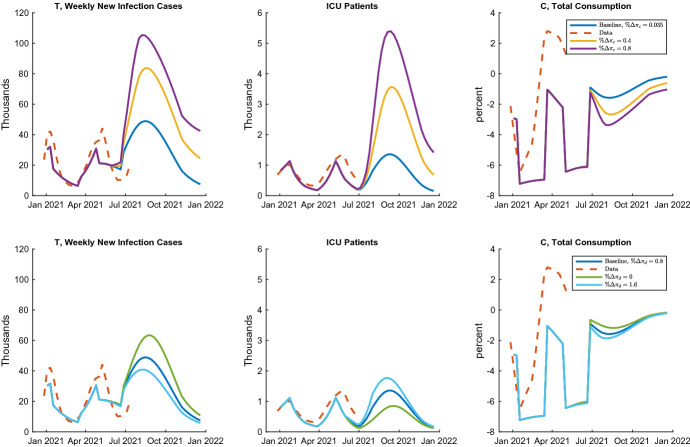
Model’s prediction under several parameter assumptions of the delta variant

Given the considerable uncertainty about the delta variant’s infection and severity parameters, it is essential to consider the robustness of the model’s prediction. Figure 8 compares the model’s predictions about the number of new infections, ICU patients, and consumption, by changing the transmission parameter $\pi _c$ and the death rate $\pi _d$, separately. Note again that, in the baseline case, $\pi _c$ and $\pi _d$ increase by $3.5\%$ and $80\%$ compared to the alpha variant, respectively. In the upper row, the rise in $\pi _c$ is changed to $40\%$ and $80\%$. Those significantly boost the infection, and then households respond and decrease the consumption. In both cases, the Japanese government may need a further soft lockdown with significant stringency. In the bottom row, I intentionally assume drastic changes in the death rate $\pi _d$ to $0\%$ and $160\%$. Compared to the case of $\pi _c$, the simulation is relatively robust. An increase in $\pi _d$ is partially offset by reducing the new infection caused by the consumption drop. In summary, this exercise shows that the delta variant’s transmissibility is crucial to both the infection and economic paths. On the other hand, the severity is relatively minor.

## Inverse lockdown

I finally evaluate an odd policy called inverse lockdown (Gonzalez-Eiras and Niepelt 2020), which encourages economic activity and spreads out the infection. At first glance, this policy is inconsistent with the well-known negative externality that people do not internalize the virus transmission to other people through their economic activities. In the literature, this is called static externality. On the other hand, dynamic externality, may cause the opposite effects, rationalizing the inverse lockdown (Garibaldi et al. 2020; Gonzalez-Eiras and Niepelt 2020; Phelan and Toda 2021). In general, the dynamic externality is an overlooked factor in individual decision problems that is about the aggregate state transitions and the continuation values. One known dynamic externality is that economic activities speed up society to converge to herd immunity. When this is not internalized, people tend to stay at home more, slowing down the convergence.

The dynamic externality may offset or dominate the static one. In the early phase of the pandemic, the static externality dominates. Given the exponential increase in $I_t$, the spread of infection causing static externality is multiplied. However, in the convergence phase, this exponential effect is diminished. On the other hand, the increase in $R_t$ is accelerated. Households should boost economic activity to accelerate the rising trend of $R_t$, but this factor is not internalized in their decision makings. Therefore, the government has an incentive to encourage household economic activity during the later periods of the pandemic. Farboodi et al. (2021) show that the optimal policy should be enduring after the pandemic’s peak, to keep the effective reproduction number above 1. Note that the dynamic externality in this paper is mainly considered *during* the periods of vaccine distribution. Due to the rapid increase in $R_t$ the dynamic externality may become even more significant. In this sense, the focus of this paper is different from a number of other papers that have suggested more severe lockdowns under the stochastic *future* arrival of vaccines (Garibaldi et al. 2020; Farboodi et al. 2021; Phelan and Toda 2021). Moreover, Makris and Toxvaerd (2020) and Gonzalez-Eiras and Niepelt (2020) study the deterministic arrival of vaccines and derive the optimality of inverse lockdown.

Inverse lockdown here is introduced as a subsidy to goods associated with infection. This policy additionally reduces $\mu _{t}$ by 1% from the baseline case for four consecutive weeks. It is subject to the static government budget constraint, financed by a lump-sum tax $B_t$:

$$\begin{aligned} B_t(S_t+I_t+R_t) = 0.01 \times [S_t c^s_{1,t}+(I_t+R_t)c^i_{1,t}]. \end{aligned}$$

Note that if there is no pandemic, this subsidy must reduce the economic welfare. It is because the pre-pandemic equilibrium of this economy is efficient. This policy is always distortionary; otherwise, the pandemic induces the dynamic externality.

**Fig. 9:**
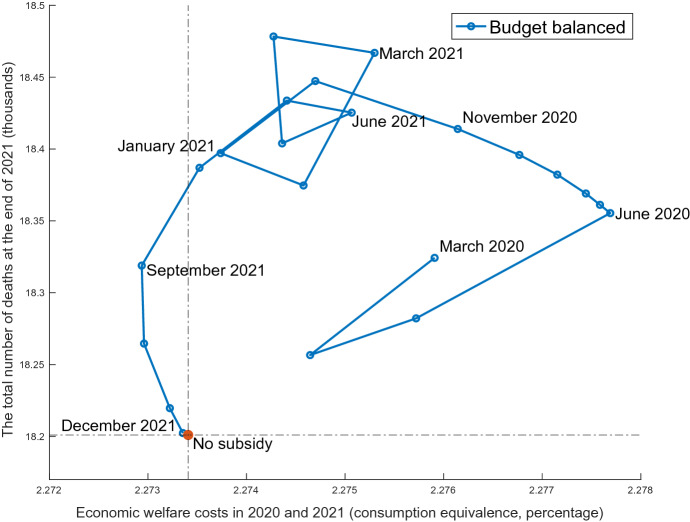
Inverse lockdown

Figure 9 plots a pandemic possibility frontier between the number of deaths at the end of 2021 and the economic welfare costs.^29^ The latter is evaluated by Eq. (23), where the time ranges between the second week of January 2020 and the last week of December 2021. Each dot indicates the result of this four-week subsidy, starting from the first week of each month. In almost all months, this policy causes damage to both the economy and health, compared to the no policy case indicated by the red dot. The Japanese government implemented “Go To Travel” and “Go To Eat” programs to subsidize face-to-face service in the fall of 2020. This model predicts negative results for both infection and the economy, although the model’s static budget constraint may be too restrictive than actual policy financed by government bonds. However, this policy improves the economic welfare in September and October 2021, months of rapid decline in the infection caused by the vaccines in Fig. 6. Since both the distortionary property of the subsidy and static externality move the economy to the right, this result implies the dominance of the dynamic externality.^30^ In actuality, this policy should not be suggested without caution, as the economic welfare has a tradeoff with the number of deaths. If Japanese society has a special preference for the economic stimulus uncovered in this model, such as the Olympic Games, egalitarian support for the damaged service industry, or long distance travel to maintain family relationships, September and October may be a better season.

## Conclusion

This paper uses a tractable SIR Macro model to examine infection from COVID-19 and economic dynamics with soft lockdown policies. I conducted quantitative exercises twice during different research periods. The first part was written in February of 2021, focusing mainly on the consequences of policy options related to the second soft lockdown, which was ongoing at the time. The results are summarized by the pandemic possibility frontiers between the economic welfare loss and total number of deaths. This model suggests that a long enough soft lockdown, or keeping the level of ICU patients stable, avoids future recurrent lockdowns. The second part was added in July 2021, to evaluate the model from an ex-post perspective. The model reasonably explains the realized paths of infection and economy; however, new COVID-19 variants caused the third lockdowns. I further project a realistic future path toward the herd immunity and evaluate the inverse lockdown under the ongoing process of vaccine distribution.

The biggest challenge of this project is studying one of the most important policy issues under the pandemic using a serious academic model (quantitative dynamic general equilibrium) while the crisis is still ongoing. The real-time project of Fujii and Nakata (2021) succeeds in various policy analyses by intentionally using a very simple model. My project complements their approach with less frequent updates but a more rigorous framework. There may be room for contributions from several directions of detailed analysis. One crucial issue is containment strategies depending on heterogeneity. For example, Baqaee et al. (2020) and Favero et al. (2020) construct realistic SIR models consisting of age and sector heterogeneities for the United States and Italy, respectively. They discussed the first lockdown exit strategies while the policies were in place in the spring of 2020. The advantage of multi-dimensional policies over uniform lockdown approaches was already well known at that time. In parallel with short-term and real-time analysis, it would be great to provide medium-term analysis of Japan with a little more structure. As a lesson for a future crisis, multiple types of quantitative analyses should be provided to allow investigation from various dimensions and robust meta-analyses.

## Figures and Tables

**Fig. 10:**
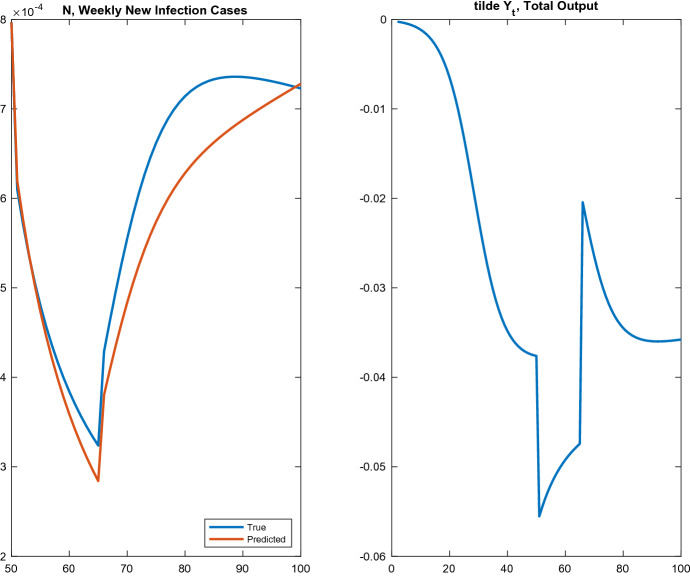
True and predicted paths of infections given the output path

## Appendix: The difference between structural and reduced-form SIR models

This appendix compares structural SIR models, including optimization and economy, as in my model, and reduced-form SIR models, such as those shown by Fujii and Nakata (2021). Both models analyze Japan’s COVID-19 infection rate, output, and policies. I discuss both the similarities and differences between these factors for policy discussion. In this appendix, I follow almost all of the same notation as Fujii and Nakata (2021). Suppose that the true state is described as this present paper’s model with no perception bias ($\forall t, \psi _t=1$). Let the percentage decline of output be ${\tilde{Y}}_t = 1 - Y_t/{\overline{Y}}_t$. I first simulate a toy economy with the soft lockdown between Periods 20 and 30 and between Periods 50 and 60. It derives the true equilibrium path of $(N_t,S_t,I_t,{\tilde{Y}}_t)_{t=1}^{100}$. Next, following Fujii and Nakata (2021), I estimate their output-infection relation parameter *h* from the true model’s results between periods 10 and 49. My goal is to evaluate the reduced-form model’s prediction using an estimation including past data and one experience of the soft lockdown.

The modified infection rate becomes

$$\begin{aligned} {\tilde{\beta }}_t = \beta _t (1-h{\tilde{Y}}_t) \end{aligned}$$

Given a unit population, the new infection is

$$\begin{aligned} N_t = {\tilde{\beta }}_t I_t S_t = \beta _t (1-h{\tilde{Y}}_t) I_t S_t. \end{aligned}$$

I estimate *h* using the nonlienar OLS, given the following equation:

$$\begin{aligned} \ln \left( \frac{N_t}{I_t S_t} \right) = \beta _0 + \ln \bigl ( 1-h{\tilde{Y}}_t \bigr ) + \varepsilon _t, \end{aligned}$$

where $\ln \beta _t$ is divided into the constant term and error term: $\ln \beta _t = \beta _0 + \varepsilon _t$. I assume that $\beta _t = \exp (\beta _0)$ for all *t* and the true path of $\bigl ({\tilde{Y}}_t\bigr )_{t=50}^{100}$. Next, given the estimated *h*, I back out $\bigl (N_t,I_t,S_t\bigr )_{t=50}^{100}$ using SIR equations.

The results are shown in Fig. 10. Overall, there is only a small difference. In general, reduced-form models capture only the correlation between infection and economy, which could include two-way causalities. However, the causality is almost solely one-way when considering lockdowns. The soft lockdowns first change economic activities and then affect infections. Thus, the estimated reduced-form correlation mainly capturing the causality from economy to infection is sufficient.

On the other hand, the estimated model predicts a slightly larger decline in new infections. The true model includes a smoothing in the event of a large shock, given the causality from infection to economy. Under the containment policy, households consume less and mitigate the infection. This effect makes less linearity under a large fluctuation than models estimated in relatively moderate periods. Nevertheless, the magnitudes look negligible.

Structural models may be required by some exercises in which causalities are crucial. For example, distributing masks is associated with a reverse causality that reduces infection first and enhances economic activity second. It improves both infection and economy, which cannot be explained by reduced-form models. In addition, structural models with nonlinear utility and production functions are necessary to capture social welfare costs. The reduced-form models tend to neglect the intertemporal distortions created by large and short-run fluctuations in economic variables and the intratemporal allocation change because of the substantial reduction in face-to-face service expenditure relative to others.

## Footnotes

1 The Japanese government enacted new legislation to levy fines against those breaking lockdown rules in February 2021.

2 The third lockdown was lifted all regions except Okinawa Prefecture. Responding to the rising infection and the Tokyo Olympics, the government imposed the fourth lockdown on Tokyo prefecture from July 12.

3 https://covid19outputjapan.github.io.

4 Covid Economics is a new attempt to accumulate academic knowledge by reacting to the quickly changing crisis. This journal briefly peer-reviews papers in a few days and publishes accepted papers online within a week, in order to balance speed and quality. The accepted papers are featured as pre-prints and can be re-submitted to formal academic journals.

5 Bethune and Korinek (2020), Farboodi et al. (2021), and Toxvaerd (2020) study abstract continuous-time models, Brotherhood et al. (2020), Eichenbaum et al. (2020b,c), Giagheddu and Papetti (2020), Glover et al. (2020), Hsu et al. (2020), and Kaplan et al. (2020) construct general equilibrium models. In addition, Kapicka and Rupert (2020) and Kang and Wang (2021) consider search markets.

6 In the Appendix, I conduct a simple numerical exercise to compare the difference between models with/without the optimization of agents. For studying containment policies, I find that agents optimizations are almost unnecessary because the causality is mainly one-way from restricting the economy to infection. However, models with agent optimizations are required if the research topic is about reverse causality, such as the vaccine’s effects on economic activities or the consequences of externality. Moreover, structural models have an advantage in social welfare measurement. The short-run fluctuations of the economic variables responding to the pandemic cause intertemporal distortions. In addition, since the COVID-19 shock is biased to the face-to-face service sector, the intratemporal allocation is also significantly distorted compared to the pre-pandemic allocation. Both factors are considered in the social welfare measurement of this paper but neglected by the reduced-form approaches in general.

7 This property is violated if the infection status declines the labor productivity. However, given the large number of patients who exhibit no symptoms, this assumption may be plausible.

8 By Walras law, the labor market clearing condition can be ignored. Moreover, the prices of the two goods are already determined as *A*.

9 https://toyokeizai.net/sp/visual/tko/covid19/en.html.

10 This assumption does not perfectly fit the simulation results of the cumulative number of total cases and death to data possibly because of the reporting lag, infection periods until death, and the rapid increase in the number of new infection cases from November 2020.

11 Krueger et al. (2020)’s baseline case is $\eta =10$. In my model, this makes the total consumption response to the soft lockdown too small to catch data.

12 Given the low reported number of cases due to the limited capacity of PCR tests in Japan, $\bar{\psi }=15$ may still carry a lower expectation than the potential number of infections.

13 I use only the time-series data in 2020 as the calibration target; otherwise, the parameter settings are also affected by the second soft lockdown, starting in January 2021.

14 It may be a bit higher number given the low infection rate in Japan. However, if I assume a lower number, numerical simulation fails due to a floating-point precision error. In the same week, I also introduce a $1\%$ reduction of $\psi _1$, otherwise $\psi _t$ stays at the constant number ${\overline{\psi }}$.

15 I use Dynare for the computation, following Krueger et al. (2020) and Eichenbaum et al. (2020b).

16 Kubota et al. (2021) and Kaneda et al. (2021) estimate the marginal propensity to consume using a large bank’s individual-level de-identified large bank’s account data. They provide varied results depending on definitions and data, but assume the MPC as 0.2. Given that the amount of the transfer is 100k Japanese Yen per person and the average amount of consumption is also about 100k Yen in the Family Income and Expenditure Survey, the transfer could push up consumption by 10% each in June and July. This number may be too large in Fig. 1.

17 Since the model is solved under agents’ perfect-foresight expectations, the scenarios in 2021 affect the equilibrium path in 2020 as well. However, since the model excludes the intertemporal saving decision, the 2021 plans are quantitatively negligible in the infection and economic paths in 2020.

18 As of July 2021, the Japanese government has not completed the vaccination to the elderly as declared in the original plan. Currently, the new goal is the end of July.

19 The death rate of patients under 65 is about 0.001. I choose a higher value by taking into account the elderly who refuse vaccine into account, following the estimation of Fujii and Nakata (2021).

20 In the model, each agent evaluates the value of death as sudden termination of his or her utility flow. In this sense, the individual-level costs of death are already included in the model. However, this could be different from society-level costs; for example, this excludes a family’s sadness over a member’s death. Because the normative costs vary, this paper takes a conservative approach, providing only pandemic possibility frontiers.

21 Given Japan’s significantly lower number of deaths than that of other countries, the corner solutions with the lowest economic damage may be realistic. On these pandemic possibility frontiers, the corner solutions are selected if the value of a statistical life (VSL) is less than about 240 years of annual GDP per capita under Vaccine 1 and 120 years under Vaccine 2. For a comparison, Hall et al. (2020) derive a realistic VSL of approximately 50 years, and Alvarez et al. (2020) assume this figure to be 40 years.

22 The central government’s plan is about 500 billion yen. Among local municipalities, Yokohama city, which has about 3.7 million population out of 125 million total population in Japan, plans 25 billion yen for vaccine. Thus, the total budget of all municipalities is estimated to be 850 billion yen. The total of the central and local, 1350 billion yen, is about $0.25\%$ of Japan’s GDP, which is approximately 550 trillion yen.

23 As of June 20, about 25 million people have received at least one vaccine shot, and among those, about 12 million have received two shots. In the simulations, the immunized populations are predicted to be 31 million under Vaccine 1 and 7 million under Vaccine 2. Therefore, the realized speed of vaccine distribution is between Vaccine 1 and Vaccine 2 in terms of both counted by one and two shots.

24 https://www.bousai.metro.tokyo.lg.jp/taisaku/saigai/1013388/1014072.html.

25 An untitled report written by Hiroshi Nishiura submitted to the COVID-19 advisory board of the Ministry of Health, Labour and Welfare on June 23. https://www.mhlw.go.jp/content/10900000/000796736.pdf.

26 In Japan, mRNA-1273 (Moderna) is also a popular vaccine; however, its reliable evidence about the delta variant has not reported yet. From a limited evidence of healthcare workers (Nasreen et al. 2021), there has almost no significant difference between mRNA-1273 and the other two vaccines.

27 The JCER’s monthly GDP is converted to the weekly measure by Spline interpolation.

28 The National Bureau of Economic Research (NBER) decides the period of this recession as only between February and May of 2020 in the U.S.

29 It is simulated under the baseline scenario. The result is almost robust to alternative delta variant’s parameters in Fig. 8, although minor changes are possible. For instance, if an 80% increase in $\pi _c$ is assumed, the pandemic’s convergence is delayed. In line with that, the month of minimal economic loss is also moved to November.

30 In Fig. 6, December 2021 is the month when the pandemic almost finishes. By then, the policy has almost no effect on the number of deaths and only harms the economic welfare.
